# The Long View of Language Localization

**DOI:** 10.3389/fnana.2019.00052

**Published:** 2019-05-24

**Authors:** Marjorie Perlman Lorch

**Affiliations:** Department of Applied Linguistics and Communication, School of Social Sciences, History and Philosophy, Birkbeck University of London, London, United Kingdom

**Keywords:** Henry Charlton Bastian, clinico-pathological correlation, aphasia, 19th century, higher cerebral function, lesion localization, single case, behavioral neurology

## Abstract

For British neurologists, one case was considered to represent significant evidence regarding the organization of language in the brain in the second half of the 19th century. The interpretation of its significance was based on repeated standard clinical assessment of behavioral deficits, the use of a psychological model of processing, and lesion localization to inform understanding of clinic-pathological correlation. The aphasic deficits experienced by a single case were observed and recorded by London neurologist Henry Charlton Bastian (1837–1915) over a period of 18 years and used as a demonstration of clinico-pathological reasoning regarding language function. This case was well documented in many of Bastian’s publications; presented in teaching demonstrations; included in discussions at medical society meetings and public lectures; and reported widely in the medical press. When this patient died, the autopsy findings were added to the extensive record of his language deficits. Some aspects of the size and site of the lesion were consistent with Bastian’s clinical predictions arising from his model of language processing, while others presented more of a paradox. This single case was a significant source of discussion and reflection in the medical community throughout the second half of the 19th century. Examination of various interpretations of this case reveal the assumptions regarding the functional architecture of language processing and more general theoretical considerations of how evidence from cases of acquired neurogenic aphasia can be employed in developing such models. This long view into a historical case sheds light on the challenges of clinic-pathological correlation methods in the understanding of localization of language functions which remain today.

## Introduction

Clinico-pathological correlation between particular higher cerebral functions and the localization of a putative source in a specific area of the brain is a primary methodological strategy for understanding the neuroanatomical foundations of linguistic and cognitive behaviors. This research program was initiated in the mid 19th century and implemented through the detailing of observational symptoms and verification by autopsy. This methodological strategy continues to be pursued in the present day through experimental tests and the deployment of various structural and functional imaging techniques with individuals who have acquired neurogenic disorders (Dronkers et al., 2017).

The development of models that relate structure of the nervous system to mental function has been built on single cases. Certain individuals have attained emblematic status in the neurosciences because their very existence has significant bearing on these models (Cubelli and De Bastiani, 2011). One such case in the 20th century was that of Henry Gustav Molaison (1926–2008), who, after a unique neurosurgical intervention, assisted in experimental research throughout his life to provide fundamental evidence regarding memory systems (Squire, 2009). The valuable contribution of such cases may be attributed to the particular purity or severity of behavioral symptoms, the stability of the clinical picture, and/or the willingness of the individual to collaborate over an extended period of time. An additional aspect is the personal qualities of the investigator driving the sustained pursuit of evidence and applying it to a developing theory.

Paul Broca’s (1824–1880) interpretation of the autopsy findings of Louis Victor Lebornge (Broca, 1861) established the clinico-pathological method which remains fundamental to neuropsychology (Lorch, 2011). This case was widely used as evidence for the theory that acquired disorders of language production were related to the existence of damage to a limited region of the frontal cortex. In the present article, another 19th century case is examined that was also a significant contribution to contemporary debate about behavior and brain relations which has not received consideration in the current literature. The individual, referred to here as Thomas A., was observed and recorded by the London neurologist Henry Charlton Bastian, FRS (1837–1915) over a period of 18 years from 1878 until his death. This late 19th century case was well known to the medical community for several decades and was viewed as a significant contribution to the understanding of the organization of language in the brain.

From the 1860s onwards, Bastian was physician at University College Hospital (UCH) and the National Hospital for the Paralysed and Epileptic in Queen Square (QS) in London. He was a leading figure of the medical community, prominent teacher of generations of medical students, and a major contributor to the neurological literature regarding sensation, perception, and movement, in addition to his work on aphasia. Extensive investigation has revealed detailed evidence concerning the high profile that Bastian’s patient had contemporaneously. This case was documented and described in many of Bastian’s publications throughout this period. Thomas A. was also presented at Bastian’s lecture demonstrations on aphasia to London medical students for over a decade. Bastian included discussions of Thomas A. in various medical society presentations and public lectures, which were the subject of review and commentary in the medical press. When Thomas A. died, his autopsy findings were presented at a medical society meeting, and the clinico-pathological correlations were considered in several subsequent publications. The case of Thomas A. was a significant source of discussion and reflection in the medical community throughout the second half of the 19th century regarding clinical symptoms, models of processing and localization of language functions.

Although Broca had established the correlation between an acquired impairment in spoken expression and a lesion in the left third frontal convolution (Broca, 1865), counter examples began to appear almost immediately (e.g., Tuke and Fraser, 1872). The relation between symptoms and lesions continued to be debated into the 20th century. In 1939, Théophile Alajouanine (1890–1980) and colleagues expressed the opinion that “in the study of aphasia facts have so little independence from theories that not only their structure, but their very existence is debatable” (cited in Gianotti, 1999, p. 135). Forty years later, Guido Gianotti highlighted the “double valence” of the aphasic syndrome, being clinical and theoretical on the one hand, and nosographical and physio-pathological on the other. Analysis of Bastian’s approach to understanding the nature of Thomas A.’s pattern of difficulties indicates his full appreciation for this duality. The present-day challenges of mapping language functions onto their neuroanatomical foundations gives Bastian’s historic case renewed value and potency.

## Clinical Context

### Bastian’s Clinical Career

**Figure 1:**
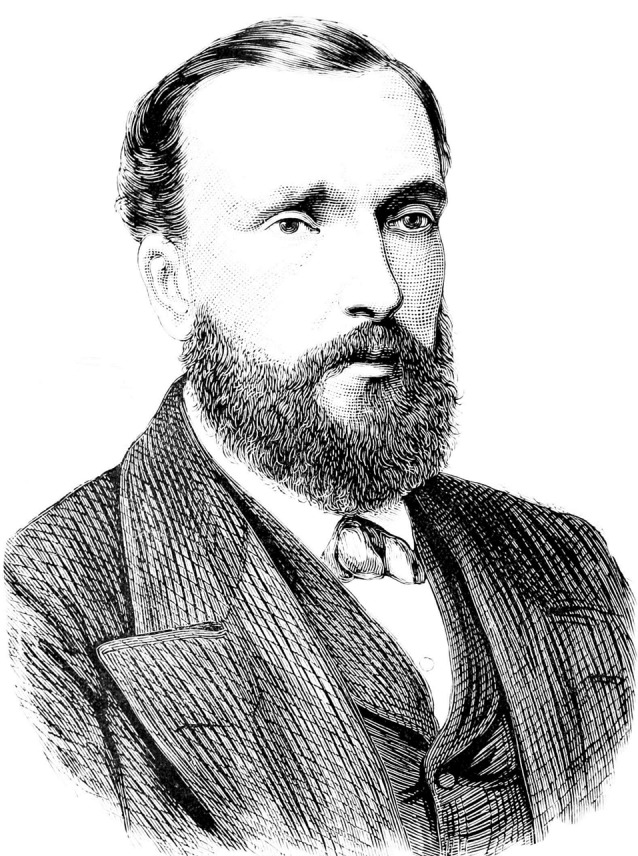
Portrait of Bastian (1875).

Henry Charlton Bastian was born Truro, Cornwall and initially pursued studies in the natural history (Anonymous, 1875b). He studied medicine at University College London (UCL) from 1859 to 1866. Two years later, at the age of 31, Bastian was elected a Fellow of the Royal Society, by Charles Darwin FRS (1809–1882) amongst others (Figure 1. Portrait of H. Charlton Bastian. Frontispiece in *The Popular Science Monthly* 1875–6, Volume 8). The polymath Herbert Spencer (1820–1903) supplied one of the testimonials for Bastian’s first medical appointment as a lecturer in pathology at St. Mary’s Hospital, Paddington. This provides a view of Bastian’s particular intellectual qualities: “… Dr Bastian has impressed me with the extent of his acquirements and the philosophical character of his intellect … to be a diligent and careful observer, as well as one who has in view the higher ends of science, to which the accumulation of special facts should be subservient” (Spencer, 1867). This attests to Bastian’s rigorous approach to evidence which would become a crucial feature of the case of Thomas A. discussed below.

After a brief period, Bastian moved to UCH and held clinical posts there and at QS continuously from the late 1860s until his retirement in the 1910s. He was a founder council member of the Neurological Society of London (f. 1886), and President in 1892. Bastian taught generations of students over four decades (1867–1907) at both at UCL and QS and his lectures were regularly published in medical journals. He also wrote dozens of original articles and books on a range of neurological topics which were widely read and reviewed.

### Bastian’s Early Contribution to Aphasia

Bastian’s early contribution to aphasia research was a brief report of a single case in the *British Medical Journal* (*BMJ*) (Bastian, 1867), but he made a significant theoretical contribution to aphasia research with his long article “On the various forms of loss of speech in cerebral disease” (Bastian, 1869b). In this article, he defined three types of impairment: “Aphemia,” “Aphasia,” and “Amnesia Verbale.” This original classification distinguished between motor speech, language, and multimodal disorders. Bastian also included the novel variable “degree of severity” to account for differences in clinical symptoms between patients in each type.

Bastian emphasized the relation of memory to four different sensory-motor modalities: speech, writing, reading, and listening. He described “Sensory Aphasia” (later referred to as “Word-deafness”) 5 years before Carl Wernicke (1848–1905) published his own model (Wernicke, 1874). His early and continuing contributions to the theoretical and clinical aspects of aphasia appear to have been neglected by contemporary French and German writers on the topic to the dismay of British proponents (De Watteville, 1885), and there were continued calls for Wernicke’s aphasia to be renamed “Bastian’s sensory aphasia” (Mott, 1917). Bastian presented his analysis of the functions of language as ultimately having a neurological basis, but his model was at the psychological level of description, with modality-based centers connected in a diffuse and interblended association network. John C. Marshall (1939–2007) considered Bastian’s visionary model to be an early form of parallel distributed network (Marshall, 2001).

After his initial work on aphasia classification, Bastian went on to consider aphasia diagnosis with regard to clinical symptoms and assessment procedures (Bastian, 1875). He described how to elicit performance from all sensory-motor modalities in order to systematically identify both impaired and spared functions. In this, he was at the forefront of systematic testing for aphasic patients (Lorch, 2013). His stated aim was to achieve “regional diagnosis upon regional symptomatology” (Bastian, 1875). This was a relatively novel objective given that, at the time, the notion that distinct areas of the cortex served different functions was still contested. Bastian’s investigation of this single case provided a focus for his thinking about clinical assessment and classification, theoretical modeling of psychological functions and sensory-motor modalities, and pathophysiological relations.

An indication of Bastian’s methodological rigor, and a foreshadowing of the difficulties presented by case of Thomas A., is included in a review of this book that appeared in the *BMJ*. The reviewer comments:

Dr Bastian deserves credit for the candor and care with which he never fails to impress the possibility of error in diagnosis, the sources of fallacy which are continually present from complexity of symptoms, and from the fact that, with regard to the function of many portions of the brain, we are still in a state of doubt and uncertainty. How often does it happen to many besides students to see their diagnosis, which ought to have been correct according to the rules laid down by systematic writers, completely set aside by *post mortem* examination!

*(Anonymous, 1875a, p. 301)*.

Shortly after his book on neurological diagnosis was published, Bastian first examined the patient Thomas A. He continued to systematically assess him through frequent examinations for almost two decades. The details of this case and its status as an illustrative example for clinical reasoning are documented in numerous published and unpublished archive sources. These are evidenced in Bastian’s hospital record book notes; lectures; articles and monographs the details of which are presented below.

## The Case of Thomas A.

### Initial Description

Although there are no remaining records of Thomas A.’s initial admission to UCH in 1878, as all case notes from this period were destroyed by the hospital, a detailed description is recorded in Bastian’s book *The Brain as an Organ of Mind* (1880). During Thomas A.’s first in-patient admission to UCH, Bastian recorded six examinations in a 1 month period. The 42-year-old tinplate worker had suffered an attack 3 months earlier without convulsions or loss of consciousness. He developed sudden right sided paralysis and “his speech was found to be almost lost” (Bastian, 1880, p. 642).

On admission, he had regained some ability to move his right limbs but had diminished sensibility. There was slight right facial paralysis and some deviation of the tongue to the right. Sight and hearing were unimpaired. Bastian determined that the patient recognized but could not name common objects; he rejected incorrect and accepted correct names when presented. He had some articulatory difficulty in repetition. His counting to 12 was good initially but became more impaired towards the end of the series. When asked to read aloud, the spoken forms bore no resemblance in sound or length to the printed form. He was also unable to write to dictation.

A month after admission, Thomas A. had suffered two slight “fits” with some increase in the severity of paralysis (In the 19th century, the term “fit” simply indicated a sudden attack which was not necessarily epileptic in nature). In his final examination before being discharged in May 1878, Bastian records that while Thomas A. was still unable to read aloud, name letters or write to dictation, his understanding of a newspaper article was indicated through gestures, and his accuracy of pronunciation in repetition had improved somewhat (Bastian, 1880, p. 642–5). This documents the patient’s onset of illness, initial early recovery of some functions and a second event.

### Examination 1 Year Post Onset of Illness

Bastian saw Thomas A. once in 1879 at QS, a year after the onset of his illness. A 14-page entry in his hospital case book records Bastian’s examination of Thomas A (Bastian, 1879). These notes provide evidence of Bastian’s assessment questions and the patient’s responses, including the specific stimuli employed and the patient’s handwriting. In recording the history of illness, Bastian notes that at onset of illness his only speech was the recurring utterance “Don’t leave me.” After some time, more spontaneous speech developed so that he could use “yes,” “no” and “never mind” appropriately, never used an oath, and could count to 100. Bastian records that after his initial admission to UCH, the patient had been sent to the Convalescent Hospital (founded 1864) at the seaside town of Eastbourne. In providing the history of illness, his wife reported that once after answering the door her husband said, “Mrs Foster will call again tomorrow,” but could not say a word of it again when asked.

Upon examination, Bastian found mild signs of right hemiparesis and sensory loss, affecting the leg more than the arm or face. In answer to the question “How are you?,” the patient was recorded as saying “fee–no–rez–ry–no–pri–pri–no–very–no.” When asked to name an inkpot he said “foo–oh,” and made additional series of attempts: “book–pook–no–the–pup–pup–I can’t.” He successfully named a book and pen, but not blotting paper. Requests to repeat produced similar responses, some accurate but others being strings of what would now be termed phonemic paraphasias. He could follow simple spoken but not written commands such as “open your mouth,” nor could he read them aloud, producing jargon instead.

### Bastian’s Patient Demonstration of Thomas A. to Students

At some point after his initial period of recovery, Thomas A. began to feature in Bastian’s presentations on “Defects of Speech” to the UCL medical students. Details of one of these lecture demonstration have been found in an archived notebook of the student William Dobinson Halliburton (1860–1931) (Halliburton, 1882). Halliburton later became a house physician in UCH serving under Bastian, and subsequently became Chair of Physiology at King’s College London from 1890 to 1923. Extracts of his notes on Bastian’s lecture were later recapitulated in Halliburton’s *Handbook of Physiology*, the leading textbook from 1896 to 1928 (Anonymous, 1950).

Halliburton records Bastian’s discussion of three different types of language impairment: Aphemia, Aphasia and Amnesia Verbale. Aphemia is defined as a difficulty of utterance or articulation, while it is noted that Aphasia may be compounded with Aphemia and Agraphia. In Bastian’s model, literate people were considered to have both an “Auditory Word Center” and a “Visual Word Center.” Aphasia may also be found together with Amnesia Verbale. The latter is considered a defect of associations of “ideal things” with ideas of words. Bastian points out that in some such patients the ability to repeat words may be preserved.

With regard to lesion localization, Bastian’s teaching reflected his acceptance of the significance of various case reports where speech impairment and a lesion in Broca’s area were not consistently or exclusively found. The student records that Aphasia “is said to be due to lesions of the third left frontal convolution: this is abundantly confirmed in the typical cases, but one must admit that lesions in other parts lead to the same conditions” (Halliburton, 1882). In Amnesia Verbale, the defect may be caused by impairment to either the centers or disturbance of the fibers of communication from the posterior end of the Sylvian fissure.

Regarding the assessment of patients with language difficulties, Bastian instructs students to look for the same six things in all cases. These are linked to aspects of his network model. Halliburton records the following set of principles:

(a) ability to understand spoken words… If the patient cannot, the auditory word center itself deranged.
(b) can the patient repeat sounds or words when requested. This tests the emission fibers from the auditory center. An aphasic person cannot do it, as it is here he is damaged.
(c) can the patient write from dictation: the sound passes into the auditory center then in order to write, it must pass thence to the visual center; then it passes out from the visual centers. This tests the fibers connecting the two centers… Then there are the same three things for the visual center…
(d) does he understand, and can he point out printed letters, and words, the patient not being blind: can he read to himself; this tests the visual word center.
(e) can he copy written words: or more complex still change printed into written words: neither can be done if the channel from the visual centers out are not intact.
(f) can he read aloud: name objects: name printed letters. This is just the opposite to writing from dictation. The impression goes into the visual word center, across to the auditory word center and off from the auditory word center.

In Halliburton’s notes it then says:

“Case Thomas A.: who can do all but (e) and (f). So his connecting fibres are damaged”

*(Halliburton, 1882, p. 255–7)*.

Halliburton also took notes on Bastian’s Lecture on Amnesia Verbale, in May 1883. There he records an examination of Thomas A. who is described as having a defect in the Auditory Word Center and in both commissural tracts. He had “no power” of spontaneous speech, except for a few words; “yes” and “no,” and when hungry “dinner” and “tea.” He could not name objects. He could repeat any word after another person but had some difficulty of articulation (Aphemia). He has no spontaneous writing. He can copy written words with his left hand, but not change printed characters into script. He could not read aloud, although he understands what he reads. After presenting a second case, Halliburton records Bastian’s conclusions that the lesion is probably at the posterior end of the Sylvian fissure, likely due to a hemorrhage, as sensibility and motion on the right side are also affected. This presentation of clinical findings corresponds to Bastian’s earlier descriptions, indicating a stable picture of symptoms. It also is a consistent interpretation of the language deficits by way of his psychological model and neurological predictions.

### Subsequent Discussion of Thomas A. in Bastian’s Books and Published Lectures

Three years after the recorded appearance of Thomas A. at his UCL lecture demonstration, Bastian published the clinical textbook *Paralysis, Cerebral, Bulbar and Spinal: A Manual of Diagnosis for Students and Practitioners* (Bastian, 1886). Bastian includes a “Schema for the Examination of Aphasic and Amnesic Patients” which represents one of the earliest to be published. It is based on the framework captured in Halliburton’s notes detailed above. Bastian stresses that it is necessary to test each patient systematically using the same set of questions because there is such variability of deficits found and how they are manifest in different modalities. He argues that the determination of the nature of combinations of symptoms will lead to a diagnosis of the underlying difficulties, and identify the nature of compromise to the system. It should be noted that Bastian’s testing of Thomas A. began during the time that Bastian was developing his assessment battery for aphasia that was subsequently published in this book. Evidence found in the QS case note records suggests that Bastian typically employed the same set of questions in examining every patient with aphasia each time he saw them, including Thomas A. This was motivated by Bastian’s methodological strategy to standardized the description of spared and impaired abilities in all four modalities in order to test his model of aphasia, and ensure the ability to validate observations (Lorch, 2013).

One indication of the impact of Bastian’s consideration of the significance of Thomas A.’s case can be found in a report of a case of “amnesia” for words published in *Brain* (Dingley, 1886). The author, Dr E.A. Dingley (birth and death dates unknown) had been a classmate of Halliburton’s at UCH 1880–1883. Dingley’s case is assessed with Bastian’s method and analyzed in terms of Bastian’s model. The author states his intention that this case serves as a useful comparison to Bastian’s case of Thomas A. who is indirectly referred to by his symptoms.

The following year, Bastian gave a lecture on aphasia at the British Medical Association (BMA) Meeting held in Dublin in his capacity as Vice-President of the Medical Section. His place on the program was highlighted in announcements, noting “… (Bastian’s) writings, on this subject especially, have won for him so high a place among neurologists” (Mapother et al., 1887, p. 201). Bastian’s presentation entitled “On Different Kinds of Aphasia, with Special Reference to their Classification and Ultimate Pathology” was subsequently published in the *BMJ* (Bastian, 1887). Here, Bastian includes another description of Thomas A. who he refers to as well known to many students at UCH. He again states that Thomas A.’s main impairment in “intellectual expression” is “referable” to a lesion involving the connections between the Auditory and Visual Word Centers. In his lecture at the BMA meeting, Bastian includes a plea for all clinicians to systematically collect autopsies for their clinical cases whenever possible; something he and his QS colleagues had been lobbying for for decades. As early as 1864, his lifelong QS colleague John Hughlings Jackson (1835–1911) stated a view that Bastian also adhered to:

“It is obvious that in the physiological study of the functions of convolutions of the brain, an autopsy is the only trust-worthy means of getting precise information. Yet it is equally obvious that a great deal of clinical work must be done beforehand if our pathological facts are to have any precise value as physiological evidence”

*(Jackson, 1864, p. 389)*.

With regard to Bastian’s model of the clinico-pathological correlation in aphasia, he repeatedly warned of the difficulty in determining from the clinical evidence whether the underlying defect is caused by a lesion in a center or connecting commissures. He suggested that while the post-mortem examination should present the possibility of resolving this in cases of well-described patients, the current body of evidence was inconclusive. This was due to a lack of adequate clinical descriptions of the exact nature of the speech defect and instances in which there was an extensive area of damage or more than one lesion found at autopsy.

Several years later, Bastian again presented Thomas A. in a postgraduate lecture reported in the *Lancet* (Bastian, 1890). The objective was to instruct students on the systematic testing of aphasic patients. He describes Thomas A.’s symptoms in detail and again links them directly to his model of functional cognitive architecture:

… Looking first to the auditory word centre with its ingoing and outgoing fibres, we find that his hearing is good and he understands readily all that is said to him. His voluntary speech is very limited… He cannot repeat short phrases, but can repeat simple words…though he often pronounces them badly owing to the existence of some amount of aphemia. If we look now at the visual word centre with its ingoing and outgoing fibres, we find that his sight is fairly good…he can read …so as to understand what he reads…he is no more able to write spontaneously than to speak spontaneously. However, he can copy…and work easy addition…Two other disabilities remain to be mentioned which show that the two commissures between the visual and the auditory word centres have been damaged in some part of their course; these are (1) that the patient cannot name at sight or read aloud even a single letter, nor (2) can he write a word or even a single letter from dictation

*(Bastian, 1890, p. 1163)*.

Although his performance on assessment is consistent with previous descriptions, Bastian now describes Thomas A.’s difficulty as “commissural amnesia.” The use of this term is linked to Bastian’s hypothesis of damage to the commissures between the auditory and visual word centers which would account for his difficulty in naming, reading aloud and writing to dictation. However, Bastian clearly states that it is not possible to extend this psychological description to the neurological level. With regard to the actual localization of neurological damage causing these symptoms, he says that the precise topographical location of these commissures is something for future research to discover. In this lecture, Bastian emphasizes the point that there is no clinical difference between aphasia produced by a lesion in Broca’s area and that by a lesion in any part of the commissure connecting it with the Auditory Word Center which is situated at the posterior end of the Sylvian fissure (Bastian, 1890).

In 1894, when Bastian expanded and revised his 1883 entry on “Aphasia” in *Quain’s Dictionary of Medicine*, he includes a description of Thomas A. anonymously. He identifies Thomas A. simply as the case described in his 1880 book that had been under his observation since, which may be taken as indirect evidence that readers were familiar with the individual. This case is presented as an example of the clinical picture of an instance where the lesion disrupted the function of commissures rather than centers (Bastian, 1894).

### The Post-mortem Findings

Bastian’s plea for the reporting of systematic clinical assessment results to advance understanding of cortical function disorders reflected his lifelong practice as an experimental scientist. Equally, he insisted on the need for post-mortem data to confirm proposed clinico-pathological correlations. His conviction about the critical value of autopsy evidence is signaled in the resolution he put to the QS Medical Committee meeting (seconded by David Ferrier (1843–1928) on 13 October 1890 (chaired by John Hughlings Jackson) (Anonymous, 1878–1899). Bastian outlined recommendations for a consistent method of reporting the name of the patient, diagnosis, name of physician, date of autopsy, and date of completion of records in the Hospital’s casebooks. Bastian’s stated motive was to facilitate the cross-referencing of autopsies with patient notes to verify his clinic-anatomical model of language and related cognitive functions. This call for the collection of autopsy data in aphasic cases was brought to wider public attention by an article in *Brain* by Dr E. Shaw [possibly identified as the London Pathologist Ernest Henry Shaw MRCS (1867–1956)]:

I would urge that in the recording of these cases, the clinical phenomena should be investigated by means of some such schema as that published by Bastian…and that the site of any lesion discovered by post-mortem should be accurately recorded…for it is only by the greatest possible accuracy in such details that contradictions in apparently ascertained facts are to be avoided, and a mass of accurate details be accumulated, from which the factors governing these phenomena may be safely deduced

*(Shaw, 1893, p. 513–4)*.

This clearly credits Bastian as the prime source of assessment practice and restates the need to pursue clinico-pathological correlation evidence in order to further theoretical understanding of the nature of aphasia.

**Figure 2:**
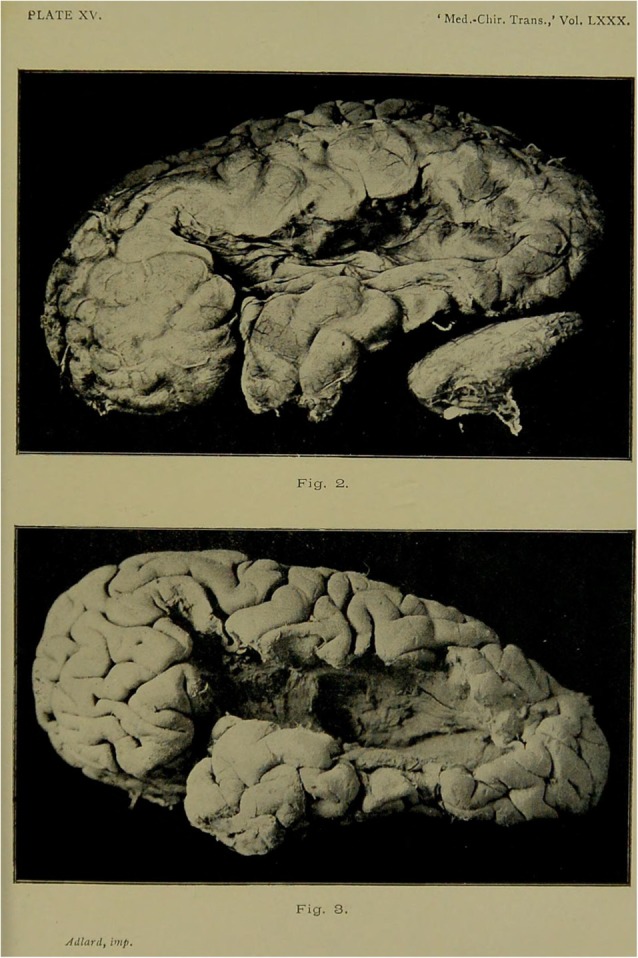
The brain of Thomas A. before and after removal of the membranes.

Hence, when Thomas A. died 3 years later from another apoplectic attack, the autopsy was duly carried out by Dr James Samuel Risien Russell (1863–1939) at QS. Bastian then presented the complete case at the Medical and Chirurgical Society which was subsequently published in the *Transactions* (Bastian, 1897b). Russell described evidence of an old softening which resulted in atrophy of the brain in the distribution of the left middle cerebral artery. While the second frontal convolution was involved, Broca’s area was spared. The upper part of the ascending frontal and parietal convolutions were also intact. The angular and supramarginal gyrus were destroyed but almost the whole of the superior parietal convolution was undamaged. The atrophic area extended posteriorly to the middle occipital convolution. The superior temporo-sphenoidal convolution was destroyed except for the anterior one third (4 1/2 cm). The middle temporo-sphenoidal convolution was also partially damaged, but the anterior portion was intact (5 cm), as was a portion of the posterior inferior portion of this convolution [Figure 2. The brain of Thomas A. before (above) and after the removal of the membranes (below); Bastian, 1897b, p. 88]. When the brain was hardened and further examined the posterior segment of the left internal capsule and most of thalamus had atrophied; anteriorly, this extended to the corpus striatum. All other portions of the left cortex were found intact, as was the cerebellum. The cause of death was determined to be the occlusion of the right middle cerebral artery but there was no evidence of older softening in the right hemisphere that had occurred before this event.

Bastian felt that in light of these autopsy findings it was necessary to substantiate the clinical symptoms. He felt this could be approached objectively as, in addition to being documented in Bastian’s own case notes, Thomas A. had been frequently examined by numerous UCH house physicians and demonstrated to students over a period of years. Bastian reports that after his initial period of illness the patient’s speech remained unchanged for the next 18 years as verified by the independent examinations recorded by Dr Boyd Joll (1878); Dr Charles Beevor (1879); Dr William Halliburton (1882); Dr Sidney Martin (1883); Dr Marc Rüffer (1886), Dr R. H. Castellote (1894–5); Dr Harold Way (1895–6).

**Figure 3:**
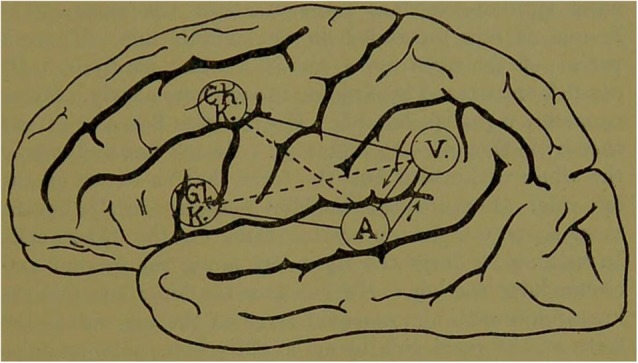
Bastian’s model, first proposed in the late 1860s, reproduced in his final case report of Thomas A. in 1897.

Apart from the two initial “fits,” and some reported intermittently for the next 5 years, his medical condition was stable. Bastian also notes that in Thomas A.’s final 2 years of life, there was some worsening of his right sided sensory-motor signs. However, the nature of his language impairment remained unchanged after the first 18 months. Although Thomas A. was described as having had chronic aphemia and aphasia (with good single word repetition and no word deafness) there was no lesion found at autopsy in Broca’s area. While this finding may have been surprising to some of his colleagues, Bastian had repeatedly suggested that an individual could have difficulties in expressive speech without having a lesion in the center itself. In discussions of Thomas A. during his lifetime, Bastian had indeed predicted that the lesion would be more posterior due to the interruption of fibers connecting the auditory and visual word centers with those more anterior centers responsible for spoken and written expression [Figure 3. Bastian’s model (Bastian, 1897b, p. 67)].

During Thomas A.’s lifetime, Bastian had repeatedly discussed the problem that in some cases it may not be possible to differentiate between the effects of damage to the centers themselves and the damage to fibers connecting them to other centers. Nevertheless, there were some aspects to the lesion localization which Bastian found puzzling. Thomas A. could not read aloud but had intact reading comprehension (no Word Blindness). His lesion was found to include the supramarginal and angular gyrus which Bastian had posited as the area responsible for what he referred to as “Visual Word Forms” (as distinct from some current uses of this term). In addition, he appeared to have good auditory comprehension (no Word Deafness) but his lesion included some but not all of the posterior superior temporal gyrus.

Bastian reviewed possible explanations for what he considered to be aspects where there was a lack of clinic-pathological correlation in a highly objective fashion. Moreover, he openly invited his peers to offer alternative interpretations. There was a discussion that ensued after Bastian’s presentation of the case at the Royal Medical and Chirurgical Society, and in a subsequent session 2 weeks later, which were reported in the *BMJ* and *Lancet*. Bastian suggested the possibility that the damage viewed at autopsy was a consequence of thrombosis which had been progressive, and the lesion had gradually enlarged over the patient’s life. To support this conjecture, he pointed to the evidence that Thomas A.’s paralysis and hemianesthesia had increased in later years. In addition, Bastian raised the possibility that the right hemisphere may have played a part in the recovery of function because of its role in language and memory. This was another point that he had considered in his earlier theoretical writings. Interestingly, from the 1870s onwards the right hemisphere had been widely considered to contribute to the recovery of language function (Finger et al., 2003; Hellal and Lorch, 2007). However, this suggestion was not widely accepted by those involved in the discussion of Thomas A.’s case at the end of the century. No successful alternative explanations were offered by his fellow discussants to reconcile the clinical symptoms and the pathological findings.

Following the publication of the case presentation, there was a very positive editorial notice on the case entitled “The Pathology of Aphasia” in the *Lancet*. This was reprinted verbatim in many international medical journals (e.g., in the *Massachusetts Medical Journal*, Anonymous, 1897). The editor suggested that “those who have followed Dr Bastian’s luminous teaching on the subject of aphasia will remember this case as frequently made use of during the patient’s lifetime to demonstrate peculiar speech defects” (Anonymous, 1896, p. 1776). The point the editorial emphasized was that Bastian had predicted that a posterior perisylvian lesion was responsible for Thomas A.’s expressive aphasia. That this was found at autopsy was seen as strong evidence in support of Bastian’s “commissural” model, and his long held view that this would account for the numerous cases of aphasia which had been presented as counter examples to Broca’s localization over the years. The editor lauded Bastian’s carefully observed case, highlighting its significance not only for aphasia but also for its contribution to physiology and psychology in general. The editor concluded that the areas of the brain involved in the understanding and expression of ideas in words was still undetermined. It was acknowledged that interpretation of the multiple aspects of clinico-pathological findings in this case was complex. Bastian himself admitted that it was difficult to reconcile the absence of Word Deafness and Word Blindness given that lesion involved supramarginal gyrus, angular gyrus and much of the superior temporal lobe.

### Bastian’s Final Considerations

After Bastian had obtained the post-mortem results, he continued to discuss the case of Thomas A. It figured in his chapter on aphasia in Allbutt’s *System of Medicine* (Bastian, 1899), where he includes a discussion of his theory with regard to lesions to centers and lesions to commissures. Bastian subsequently discussed the Case of Thomas A. in his Lumleian Lectures at the Royal College of Physicians which were published in the *BMJ* (Bastian, 1897a). These lectures served as the foundation for Bastian’s most long-lasting contribution on the topic, his *Treatise on Aphasia and other Speech Defects* (Bastian, 1898). In this monograph that summarized his life’s work on the neurological and psychological foundations of language functions, he discusses Thomas A.’s case again. He reiterates the frank admission that there is some difficulty reconciling the clinical observations with the lesion found at autopsy.

**Figure 4:**
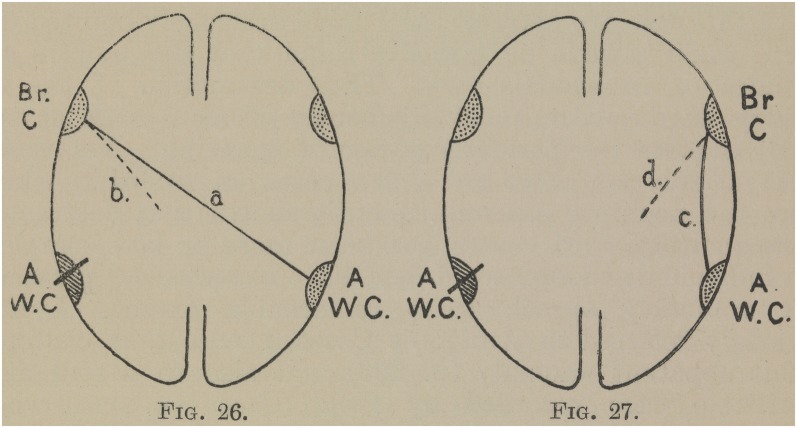
Bastian’s proposal for a possible mode of recovery from Word-Deafness after damage to the left Auditory Word Center as in the case of Thomas A. through a connection between the right Auditory Word Center and Broca’s Center on the left side (a.) and the recruitment of “internuncial fibers” from the latter (Bastian, 1898, p. 330).

In offering an explanation, Bastian raises an issue he had addressed in his writings over the decades: the nature of the developmental process of spoken language and literacy in children, and in this context, sources of individual variation in language organization. Bastian had also suggested that these aspects might need to be considered in accounts of recovery through variation in compensatory mechanisms. In his *Treatise*, Bastian raises this as a possible explanation for the unusual picture of behavioral impairment and autopsy findings in the case of Thomas A. Bastian makes this account more explicit by detailing how this individual’s ability to understand speech and print might be due to right hemisphere involvement. Thomas A. is presented in detail as “Case XCIV.” Bastian also included an illustration of possible modes of recovery from Word Deafness with two sagittal section diagrams and arrows indicating the potential compensatory networks from the posterior right hemisphere Auditory Word Center and the commissures linking this to the Broca’s Center on the left and right [Figure 4. Bastian’s proposal for a possible mode of recovery from Word-Deafness after damage to the left Auditory Word Center as in the case of Thomas A. through a connection between the right Auditory Word Center and Broca’s Center on the left side (a.) and the recruitment of “internuncial fibers” from the latter (Bastian, 1898, p. 330)]. He speculated that in some individuals, the right Auditory Word Center was more active, and had some connections to Broca’s area on the left side. This would provide a compensatory mechanism when the left auditory word center was damaged and was offered as an account for the clinic-pathological picture presented by Thomas A.

In a book notice on Bastian’s *Treatise* in the *New York Medical Journal* (Anonymous, 1898), the reviewer asserts that aphasia is one of the most difficult medical problems to solve in all of medicine. This, they suggest, is due to its inherent complexity and the lack of cases that have provided a clear clinical picture alongside a pathological description of the brain at death. The reviewer praises Bastian’s application of this scientific method to his investigation of neurological disease in general and aphasia in particular. The case of Thomas A. can be seen as a detailed empirical demonstration of his theoretical principles.

## Discussion

### Persistence and Propagation of Bastian’s Ideas

Throughout his career, Bastian made distinctions between the rationale for clinic-pathological correlation and localization of function. His model included functional centers at the level of psychological description, but he emphasized the networked and distributed nature of this functional architecture. Moreover, this did not map directly on to the location of a neurological lesion. It has been suggested by early 20th century historians of aphasiology that Bastian’s utilitarian approach to clinical aphasiology was more influential than his QS colleague John Hughlings Jackson’s in the later 19th century (Head, 1921; Weisenburg and Mcbride, 1935).

Henry Head (1861–1940), who was at UCL and QS in the late 1880s and early 1890s, referred directly to the case of Thomas A.’s in his own work on aphasia (Head, 1926). In his monograph on aphasia, Head pointed to the symptoms Bastian had described, the predictions he had made based on his model, and the difficulty presented in reconciling the post-mortem findings in disparaging terms. Head was of the opinion that what Bastian identified as the clinical condition was simply “a translation of the phenomena into *a priori* conceptions, which had no existence in reality” (Head, 1926, p. 57). Head’s remarks were made in aid of his larger program to reinstate Jackson as the premiere founding father of aphasiology by denigrating Bastian and other colleagues.

However, three decades later, Sir Gordon Holmes FRS (1876–1965) who was a physician at QS from 1906, reconsidered Bastian’s lasting contributions. He expressed the opinion that:

[Bastian was] adversely criticized by Head and others, and although he is rarely cited [today] as an authority on the subject, he certainly contributed much to our knowledge of the physiology of speech and its disturbance by disease… his rigidly scientific outlook was scarcely suited to elementary clinical practice… but the house physicians who accompanied him [on rounds] invariably profited from his comments as he examined patients and discussed their symptoms, and by his precise and logical methods in arriving at a diagnosis

*(Holmes, 1954, p. 38)*.

Bastian’s ideas had widespread influence as disseminated through the teaching he delivered to generations of London medical students and by his numerous articles in medical journals and bestselling books. It has continued to have influence throughout the 20th century. One such example can be found in the work of the Harvard neurologist Norman Geschwind (1926–1984) and his colleagues at the Boston Veterans Hospital Aphasia Research Unit, Harold Goodglass (1920–2002) and Edith Kaplan (1924–2009).

Geschwind was introduced to Bastian’s work through the training he gained in neurology at QS in the 1950s by Charles Symonds (1890–1978) amongst others (Devinsky, 2009). Symonds joined QS in 1919 and was a junior colleague of Gordon Holmes and Samuel Alexander Kinnier Wilson (1878–1937) who had both been trained by Bastian. Symonds was considered one of the major early 20th century clinicians at QS (Compston, 2009), who had developed an interest in aphasia late in his career. In his 1953 article on aphasia, Symonds aligns his views with Bastian’s ideas on aphasia rather than Head’s teachings which were the predominant view held by most of his contemporaries. Symonds conceptualization of localization of function with respect to psychological and neurological models is consonant with Bastian’s:

It seems nowadays to be the fashion in discussing aphasia to begin with the consideration of speech as a psychological function and to proceed to the analysis and classification of the dysphasias in terms of disordered thinking, with little concern for the problems of anatomy and physiology that must be involved when a disorder of speech results from local disease of the brain. This represents a swing of the pendulum away from the method of the older writers on this subject, who sought on the basis of clinical and pathological observation to classify the dysphasias in terms of the situation of the lesion and its interference with hypothetical centres and pathways serving the different functions involved… It is true that the disorders of speech function can be described only in psychological terms, but if we repeatedly observe in disease a specific disorder of speech function the occurrence entitles us to conclude that there are separate anatomical arrangements, or one might equally well call them neurophysiological dispositions, subserving the psychological function affected. This, I think, was what the more thoughtful of the earlier writers on aphasia meant when they used the word “centre,” though this term later became debased and has gone out of fashion… If we examine the clinical facts impartially we shall find, I submit, abundant evidence of the localization of function in this sense

*(Symonds, 1953, p. 1–2)*.

Although he does not directly mention Bastian as the source of these ideas, Symonds draws heavily on Bastian’s model of aphasia; and Bastian’s *Treatise* (1898) is the most cited source in the article. In his conclusion, Symonds suggests that: “unless classifications are established the opportunities occasionally offered by disease [through autopsy] for the localization of function will be missed…” (Symonds, 1953, p. 5). This statement also clearly reflects Bastian’s own rationale.

As such, much of what Symonds discusses regarding Bastian’s ideas prefigures the approach to be embraced by Symonds’ own student Norman Geschwind. It is clear to see the influence of Bastian’s theories on Geschwind’s model of disconnection syndromes and his work on aphasia a decade later. In fact, Geschwind’s original presentation of his disconnection theory (Geschwind, 1965) begins with a citation to Bastian’s work. In the Geschwind papers held at the Archives of Countway Medical Library at Harvard University, I found copious notes made by Geschwind when reading Bastian’s works (Geschwind, 1872).

Some years later, one of Geschwind’s own students, Jason Brown, reprinted Bastian’s *Treatise on Aphasia*. In the preface he commented:

For many of us who have labored for a time in the convoluted mines of aphasia, this work by Charlton Bastian has been discovered among the rubble as something of a semi-precious stone. Not only is it a particularly valuable sourcebook of the early literature, but more importantly for the contemporary student, the work has proved to be one of the most authoritative statements of a specialized account of the aphasias, the so-called disconnexion approach that has yet been published

*(Brown, 1984)*.

The *Boston Diagnostic Aphasia Examination* (BDAE; 1972) developed by Geschwind’s colleagues Goodglass and Kaplan can also be seen as a 20th century implementation of assessment techniques initially championed by Bastian. They shared Bastian’s objective of developing a systematic and robust approach to the “diagnosis of presence and type of aphasic syndrome, leading to inferences concerning cerebral localization” (Goodglass and Kaplan, 1972, p. 1). Like Bastian’s system, their test included the dimension of severity; was also intended to provide broad classifications; and provide some indications of lesion localization. However, in the late 20th century, just as in the late 19th century, there was growing resistance to such an approach by clinicians who felt that there were only a minority of cases that would successfully fit these models. This led to a rejection of the clinico-pathological correlation as an analytical tool toward the end of the 20th century. Ola Selnes characterized the problem in his review of a leading textbook on aphasia (Benson and Ardila, 1996):

Since Broca’s early attempts to correlate changes in speech and language with injury to specific regions of the left hemisphere, aphasia has been the flagship syndrome of behavioral neurology. This is the case partly because no other changes in higher cognitive functions have shown such consistent and predictable relations with the underlying brain injuries. Nevertheless, despite Pierre Marie’s effort to simplify the classification of aphasia syndromes, a complex and sometimes intimidating diagnostic system has evolved over the years. Internists, neurologists, neuropsychologists, and other practitioners are often reluctant to fit patients’ speech and language symptoms to a specific aphasia syndrome

*(Selnes, 1997, p. 143)*.

### Some Final Considerations

Current discussion about models of language processing (e.g., Hickok and Poeppel, 2004) are now being considered in light of historical models. These comparisons are typically made with respect to the so-called “classic model” which draws on the 19th century ideas of Carl Wernicke (1874) and Ludwig Lichtheim (1845–1928; Lichtheim, 1885) and their later reconceptualization by Geschwind (1970). Tremblay and Dick (2016), amongst others, reject this style of model although its use widely persists today. Their objections arising from current neuroanatomical understanding include the problematic and limited focus on the putative function of particular cortical areas such as “Broca’s area” and “Wernicke’s area,” and lack of consideration of cortical and subcortical connections beyond that of the arcuate fasciculus.

In contrast to his 19th century contemporaries Wernicke and Lichtheim, Bastian presented a more cautious view of what cortical areas and the connections between them actually entail. He tended to refer to areas and their connections by their behavioral functions, keeping open the question of the actual brain structures that might support them. Throughout his career, he tended to use his own terminology which linked sensory-motor modalities and their roles in various aspects of language processing. For example, he employed the term “glosso-kinesthetic center” in preference to “Broca’s area.” In his final major work on aphasia, his *Treatise*, Bastian bows to fashion but uses the term “Broca’s center” underscoring its functional status rather than any anatomical integrity, as in Figure 4. Bastian argued that these notional functional “centers” were of clinical utility, aiding the conceptual mapping between observed symptoms and possible sources of pathology. At the same time, he continually stressed that he did not intend this type of reasoning to be used to directly infer anatomical function. From early in his career (Bastian, 1869a) until his final publications at the end of the century, Bastian continued to insist that until physiological understanding was further developed, the nature of the relation between psychological models and their neuroanatomical instantiation was undetermined. However, he did believe that this was the ultimate goal, attainable in the future.

While there have been great advancements in these domains since Bastian’s time, some undetermined aspects remain. These continue to present challenges to the interpretation of the case of Thomas A. For example, the role of Brodmann area 22 in language comprehension disorders remains an unsettled issue (e.g., Dronkers et al., 2004). This problem still has bearing on the difficulty Bastian had in reconciling Thomas A.’s good auditory comprehension despite having a lesion in a portion of the superior temporal gyrus. Bastian had invoked individual differences and possible sources of compensation in the right hemisphere in his account. He also had acknowledged that there was limited evidence of the actual anatomy of the commissural connections that his psychological model was predicated on. In addition, Bastian’s model did attempt to capture the modality-specific representations for reading and writing and their input and output pathways. However, he had difficulty accounting for Thomas A.’s good reading comprehension, but inability to read aloud or write to dictation, given that his lesion involved the supramarginal and angular gyrus. The function of these areas and their connections to other regions also remain unsettled today (e.g., Seghier, 2013; Bouhali et al., 2014).

The development of cognitive models in neuropsychology has also been challenged by the new body of functional imaging findings. This can be seen in a recent review of the evolution of cognitive models of language function that begins in the second half of the 20th century (Price, 2018). It argues that the way forward to resolving current difficulties in explaining individual variation and the mechanisms of compensation is to combine findings from functional imaging with behavioral testing and lesion mapping of impaired individuals to develop better accounts of inter-subject variability. This would join our new techniques to Bastian’s approach which had recently been underutilized: that of rigorous clinical assessment of spared and impaired abilities, and the use of pathological findings to attempt to resolve the inherent difficulties presented by individual differences in behavior patterns.

The question regarding the methodological value of single case studies vs. group studies has also been a topic of debate in neuropsychology research. In the 1980s, this was discussed specifically with regard to difficulties in reconciling findings with regard to aphasia (e.g., Howard, 1986). While large group studies and randomized control trials became the research design of choice in the second half of the 20th century, it appears that the pendulum has swung back to some extent on this point as well. The value of single case studies to neuropsychology has been reaffirmed (e.g., Cubelli and Della Sala, 2017). Most recently, there have been attempts to address the reproducibility crisis in symptom localization with neuroimaging studies. Darby et al. (2019) propose that lesions in different locations may give rise to the same behavioral symptoms due to their connection in a common localizable functional network. This account would be consistent with the model proposed by Bastian over 150 years ago.

In summary, the defining features of Bastian’s contribution are his model of psychologically defined centers connected in multiple ways in a network, his clinical approach to aphasia standardization of assessment and the use of classification, and his reasoning through clinic-pathological correlation to understand language function in the brain. Secondarily, he repeatedly pointed out that individual variation in the picture of behavioral symptoms, and the underlying pathology that had caused them, was due in part to constitutional differences and the influences of lifetime experiences; what would now be considered as aspects of epigenetic variation, differences in maturational trajectory, and the influence of socio-cultural factors. These ideas were tested through prolonged consideration of the case of Thomas A. As such, this data-rich longitudinal study with post-mortem findings represents a significant historical source for the understanding of aphasia.

## Contribution to the Field Statement

How language functions are represented in the brain has been a topic of investigation since the 19th century. One challenge is to determine how psychological models map on to neurological systems. A second challenge is to explain the variation found in individual cases between their pattern of impairments and the pathological source. One language impaired patient was followed for almost two decades by the London neurologist Henry Charlton Bastian at the end of the 19th century. The details of this case, and its impact on the medical community, are revealed through the analysis of detailed archival sources. Its significance was based on the repeated clinical testing, interpretation of findings with a psychological model of processing, and predictions about the areas of the brain involved in language functions. When this patient died, some aspects of the brain damage found at autopsy upheld Bastian’s clinical predictions, while others presented more of a puzzle. Interpretation of this case illuminates how evidence from language disorders is used to develop models of brain and behavior relations. Consideration of this historical case sheds light on the ongoing difficulties in understanding the relations between the function of areas of the brain and their connections to the language processes they bear which remain today.

## Author Contributions

The author confirms being the sole contributor of this work and has approved it for publication.

## Conflict of Interest Statement

The author declares that the research was conducted in the absence of any commercial or financial relationships that could be construed as a potential conflict of interest.
